# Effects of Transcranial Pulse Stimulation of the Primary Motor Cortex on Motor Performance in Healthy Adults: A Randomized Crossover Pilot Study

**DOI:** 10.1002/cns.70711

**Published:** 2025-12-20

**Authors:** Penny Ping Qin, Ivan Kin‐Yeung Chak, Rebecca Lai‐Di Kan, Min‐Xia Jin, Bella Bing‐Bing Zhang, Adam Wei‐Li Xia, Tim Tian‐Ze Lin, Sharie Xiao Wang, Jian‐Hui Liu, Teris Cheung, Georg S. Kranz

**Affiliations:** ^1^ Department of Rehabilitation Sciences The Hong Kong Polytechnic University Hong Kong China; ^2^ Shanghai YangZhi Rehabilitation Hospital (Shanghai Sunshine Rehabilitation Center), School of Medicine Tongji University Shanghai China; ^3^ Department of Anesthesiology, School of Medicine Tongji University Shanghai China; ^4^ School of Nursing The Hong Kong Polytechnic University Hong Kong China; ^5^ Mental Health Research Center (MHRC) The Hong Kong Polytechnic University Hong Kong China; ^6^ University Research Facility in Behavioral and Systems Neuroscience (UBSN) The Hong Kong Polytechnic University Hong Kong China

**Keywords:** motor cortex, motor function, noninvasive brain stimulation, transcranial pulse stimulation

## Abstract

**Aims:**

We aimed to investigate the behavioral aftereffects of a novel noninvasive brain stimulation technique—transcranial pulse stimulation (TPS)—applied over the right motor cortex (rM1) in healthy adults.

**Methods:**

Thirty‐four healthy adults underwent a randomized, subject‐ and analyst‐ blind, crossover trial, receiving active TPS over the rM1 or control TPS over the vertex in two sessions 24 h apart. Motor performance was assessed using the Nine‐Hole Peg Test (NHPT) and the Simple Reaction Time Task (SRTT) before, immediately after, and every 10 min for 40 min after each session. A linear mixed model and post hoc analyses were applied to evaluate the effects.

**Results:**

No significant interaction effect (stimulation condition × time) was found. The left‐hand NHPT performance significantly improved from 10 min post‐TPS onward in both conditions (*p*s ≤ 0.002).

**Conclusion:**

A single session of rM1‐TPS does not yield significant improvements in motor dexterity compared to vertex‐TPS. Future well‐powered studies with a sham control condition and multiple stimulation sessions are needed to investigate the aftereffects using a combination of neurophysiological and neuroimaging approaches.

**Trial Registration:**

This study was registered in ClinicalTrial.gov in April 2024 (NCT06312930)

## Graphical abstract

A single session of transcranial pulse stimulation (TPS) at the right primary motor cortex (rM1) does not improve hand dexterity compared to vertex stimulation. A similar practice effect was observed for both stimulations from 10 to 40 min post‐stimulation.

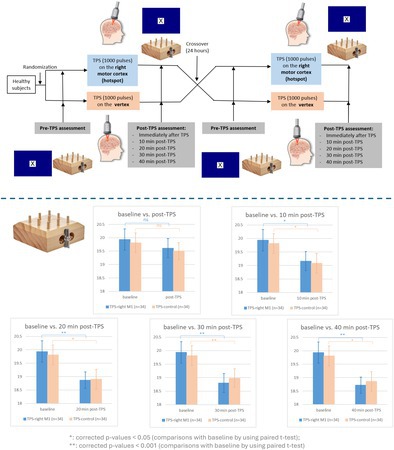

## 1 Introduction

Transcranial pulse stimulation (TPS) is a novel noninvasive brain stimulation technique that uses high‐pressure ultrashort ultrasound pulses to modulate brain function [1]. Developed from low‐energy extracorporeal shock wave therapy (ESWT), TPS influences cellular processes through mechanotransduction, promoting neovascularization, tissue regeneration, and anti‐inflammatory processes [2]. In vitro studies demonstrate that ESWT enhances the expression of vascular endothelial growth factor, brain‐derived neurotrophic factor, and endogenous nitric oxide synthase, supporting neurovascular health [3, 4, 5, 6].

TPS is a CE‐approved device for Alzheimer's disease (AD) treatment [7]. Early pilot studies reported cognitive improvements following 6–12 TPS sessions with benefits lasting up to three months, correlating with increased functional connectivity within the memory network and cortical thickness in the left superior parietal lobule [8]. Subsequent research has investigated TPS across several neurological and psychiatric disorders, including mild neurocognitive disorder, depression, Parkinson's disease (PD), Autism Spectrum Disorder (ASD), and Attention Deficit Hyperactivity Disorder (ADHD) [9, 10, 11, 12, 13]. Notably, a recent randomized controlled trial demonstrated the therapeutic and neuromodulatory effects of a 2‐week verum TPS treatment in younger AD patients [14].

Despite promising findings, most TPS studies have been uncontrolled and distributed stimulation across several cortical regions. This approach has left the region‐specific behavioral effects and the underlying neuromodulatory mechanisms unclear. To date, only three randomized controlled TPS trials have investigated target‐specific neuromodulatory and behavioral effects [7, 15, 16]. Beisteiner et al. [7] demonstrated that a single session (1000 pulses at 4 Hz) over the somatosensory cortex (S1) induced immediate neurophysiological alterations in healthy individuals, as measured by somatosensory evoked potentials. Furthermore, three sessions of S1‐TPS led to a long‐lasting enhancement in global sensorimotor efficiency one week later [16]. More recently, a clinical trial demonstrated the behavioral modulatory effects of TPS, finding that a single session (1500 pulses at 4 Hz) over the primary motor cortex (M1) immediately reduced tremor amplitude in PD patients compared to sham stimulation [15].

Since the aftereffects of a single TPS session are largely unexplored and motor function serves as an objective readout of behavioral modulation, we conducted this randomized, subject‐ and analyst‐blind, crossover‐controlled pilot trial. The aim was to investigate whether TPS over the right primary motor cortex (rM1) enhances left‐hand motor performance in healthy individuals. Based on analogous effects shown by transcranial current direct stimulation and low‐intensity transcranial ultrasound stimulation [17, 18, 19], we hypothesized that rM1‐TPS would improve left‐hand performance with aftereffects lasting 30–40 min.

## 2 Methods

### 2.1 Study Overview

The current study is a two‐visit, randomized, subject‐ and analyst‐blind, controlled pilot trial with a crossover design, conducted at the Hong Kong Polytechnic University. Thirty‐five right‐handed (as assessed by the Edinburgh Handedness Inventory score > 40) healthy adults aged between 18 and 65 years were recruited from posters posted at the Hong Kong Polytechnic University from March 2024 to December 2024. Exclusion criteria were as follows: (1) history of any neurological, musculoskeletal, or psychiatric disorders; (2) history of brain injury or cranial bone defects; (3) untreated coagulopathies (hemophilia); (4) thrombosis in the stimulated area; (5) pregnancy; (6) malignant tumor in the stimulated area; (7) cortisone therapy up to 6 weeks before first TPS session; (8) metallic objects in the head; (9) pacemakers that are not authorized for focused shock wave therapy; (10) simultaneous utilization of medication that lower the seizure threshold; (11) implanted deep brain stimulation device; (12) history of substance abuse or alcohol dependence; (13) with neuroscience or medical background. Eligible participants signed the written informed consent after the experimental procedures were fully explained. This study was performed according to the Declaration of Helsinki [20], and the study protocol was approved by the Human Subjects Ethics Sub‐committee of The Hong Kong Polytechnic University (Reference No: HSEARS20240319001‐03). This study was registered in ClinicalTrial.gov in April 2024 (NCT06312930).

**FIGURE 1:**
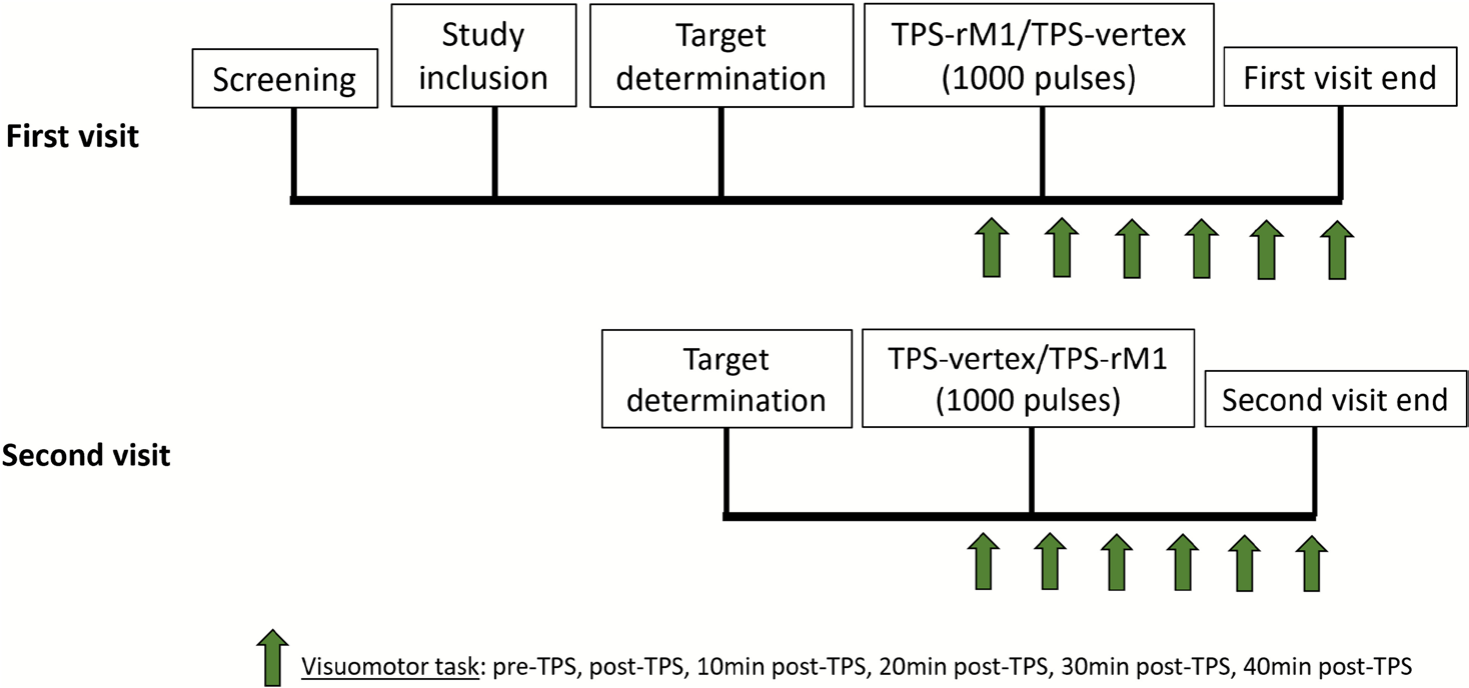
Study procedure. Screening: Phone screening or face‐to‐face screening was conducted to select eligible participants by using a screening form before the first experimental visit; Study inclusion: On the first experimental visit day, participants were included in the study, and a consent form was signed by them before the start of the experiment; Target determination: Hotspot in the right primary motor cortex (rM1) was determined by using single‐pulse transcranial pulse stimulation and electromyography. Vertex was determined as the midpoint between the nasion and inion along the midline. Both targets were determined and marked during both visits to ensure the integrity of participants' blinding; TPS‐rM1/TPS‐vertex, TPS‐vertex/TPS‐rM1: 1000 TPS pulses were delivered into rM1 or vertex during the first experimental visit, and the alternative target during the second visit; Visuomotor task: Nine‐Hole Peg Test and Simple Reaction Time Task were measured for the left hand before TPS, immediately after TPS, 10 min after TPS, 20 min after TPS, 30 min after TPS, and 40 min after TPS.

Of the 35 consented participants, 34 completed the study (18 male, 16 female; mean age = 36.9 ± 17.7 years; range = 18–65 years). One participant whose active target (the hotspot in the rM1) could not be identified by TMS was excluded from the experiment. After enrollment, participants were randomly assigned to receive a single TPS session of 1000 pulses at either the rM1 or the vertex during the first visit, followed by the alternative target during the second visit in a randomized, counterbalanced order. The two experimental visits were scheduled 24 h apart. Before each TPS session, both the active target (hotspot in the rM1) and the control target (vertex) were identified and marked on the scalp. Motor performance was assessed using the nine‐hole peg test (NHPT) and the Deary‐Liewald simple reaction time task (SRTT) at baseline and at multiple post‐TPS time points (immediate, 10, 20, 30, and 40 min after stimulation) to examine changes over time. The study procedure is illustrated in Figure 1.

### 2.2 Locating Stimulation Targets

**FIGURE 2:**
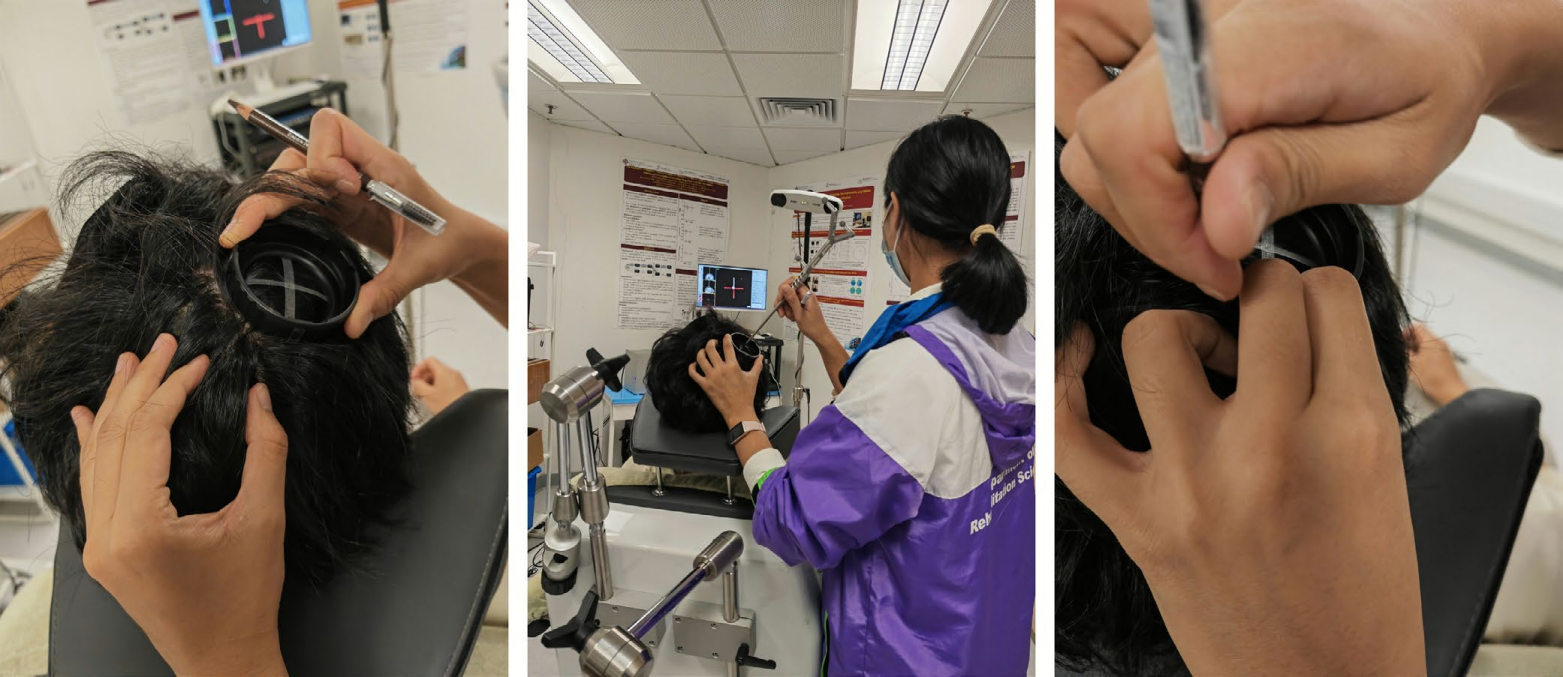
Procedure for marking the hotspot in the right primary motor cortex. Left: A circular ring removed from the TPS handpiece was used for marking, with its center indicating the TPS handpiece's focal point; Middle: The hotspot was positioned at the center of the circular ring; Right: The ring's circumference was marked on the scalp using a brown eyebrow pencil.

The active target of the hotspot in the rM1 was identified using single‐pulse TMS (MagVenture MagPro X100). A figure‐of‐eight TMS coil was positioned with its handle oriented 45° laterally and posteriorly to the sagittal plane. Starting at a low stimulation intensity, single TMS pulses were delivered at different scalp locations over the primary motor cortex. Motor‐evoked potentials (MEPs) of the left first dorsal interosseous (FDI) were recorded at rest using a pair of 25 mm × 20 mm surface electrodes in a belly‐tendon montage with the TMS device's MEP monitor. The hotspot was defined as the scalp location where single‐pulse TMS consistently elicited the largest MEP amplitude in the left FDI muscle at the lowest stimulation intensity. The location was then marked on the scalp with a brown eyebrow pencil to ensure precise TPS targeting. Figure 2 illustrates the hotspot marking procedure.

The control target of the vertex was defined as the midpoint between the nasion and inion along the midline. The marking procedure followed the same protocol as used for the hotspot. Before each TPS session, both the active and control targets were marked to minimize potential bias in the marking process.

### 2.3 TPS Parameters and Administration

**FIGURE 3:**
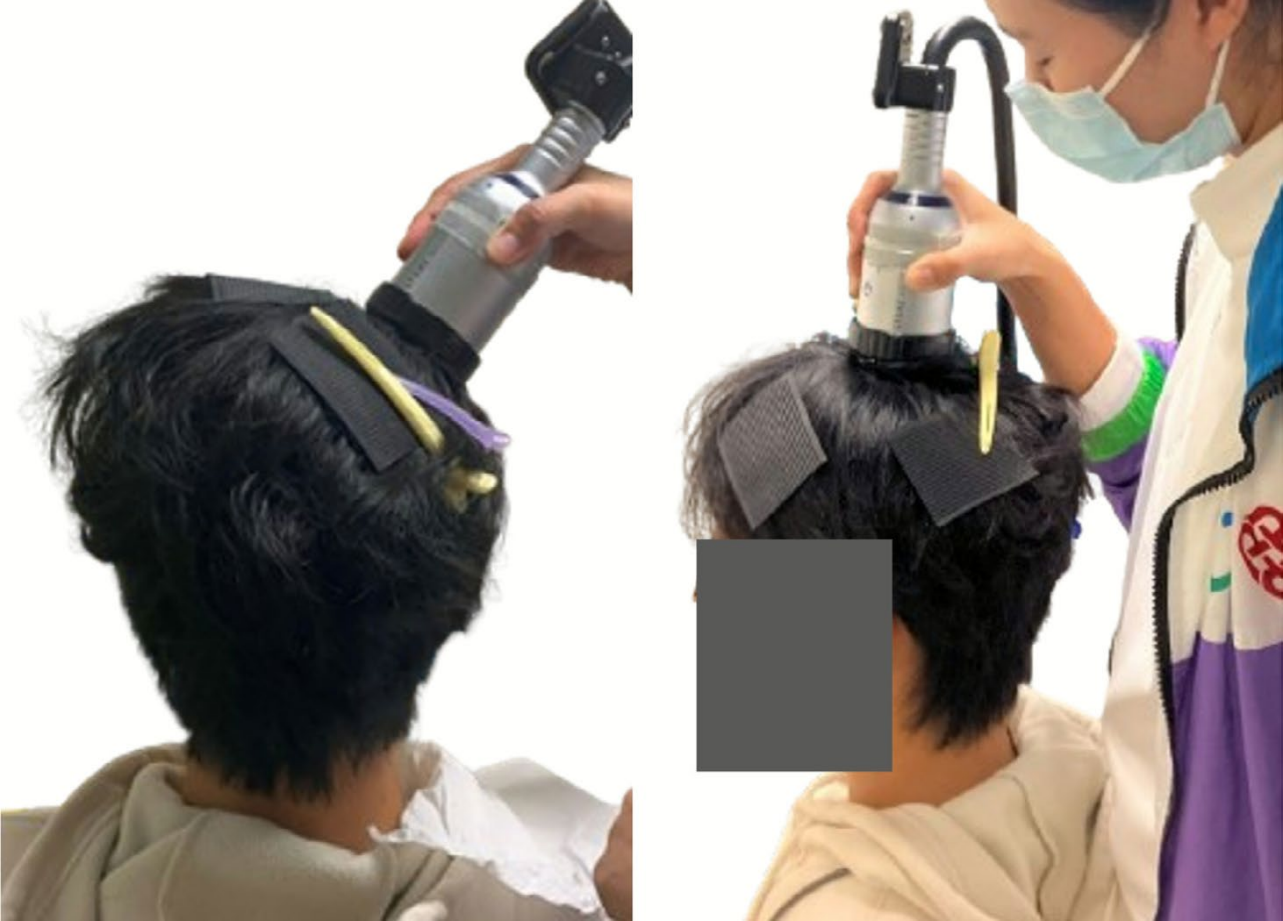
TPS handpiece positioning for (left) hotspot in the right primary motor cortex and (right) vertex stimulation. Hair stickers and clips were used to keep hair away and expose the markers.

TPS was delivered using the NEUROLITH system (Storz Medical AG, Tagerwilen, Switzerland). During active stimulation sessions, 1000 TPS pulses were delivered at a pulse frequency of 4 Hz with an energy flux density (EFD) of 0.20 mJ/mm^2^ to the hotspot in the rM1 (Figure 3, left), similar to the parameters utilized in previous research [7, 16]. The TPS pulse has a focus size of 5 mm (lateral) × 5 mm (sagittal) × 56 mm (axial) [21] and a focus depth of 43.4 mm (with a 6.6 mm stand‐off device). To ensure optimal acoustic coupling, a generous amount of bubble‐free ultrasound gel was applied to the stimulation site. The TPS handpiece, equipped with the circular ring, was carefully aligned with the marked circumference and maintained in a perpendicular orientation to the scalp throughout stimulation. Control stimulation sessions followed the same procedural setup and parameters, with the only difference being the target location—the vertex instead of the motor hotspot (Figure 3, right). An adverse event checklist was used after each TPS session to assess the side effects.

### 2.4 Behavioral Assessment

The Deary‐Liewald SRTT and NHPT were used to assess the motor performance at baseline (T0) and immediately after (T1), as well as 10 (T2), 20 (T3), 30 (T4), and 40 min (T5) following TPS. The tests were given in a fixed sequence: first the SRTT, then the NHPT.

The SRTT assesses processing speed by measuring reaction times in response to a diagonal cross appearing on a computer screen [22]. Participants pressed the space key as quickly as possible after the stimulus appeared. The stimulus remained on the screen until the participant responded, and the interval between stimuli varied randomly between 1 and 3 s. Each session included 10 practice trials and 20 test trials, performed first with the left hand, then the right. Reaction times were automatically recorded, and outliers—responses exceeding 1 s or below 0.1 s—were excluded. Two independent analysts extracted the data, calculating the mean and standard deviation (SD) for analysis.

The NHPT assesses hand dexterity utilizing the Jamar 9‐Hole Pegboard [23, 24]. The square pegboard (12 cm × 12 cm) has nine holes (7 mm diameter, 26 mm apart from each other) and comes with pegs (32 mm length, 6 mm diameter). The pegboard and a dish were placed at the participant's midline, with the dish oriented toward the tested hand (first with the left hand, then the right hand). During the test, participants took pegs one by one, placed them into the holes, then returned them to the dish as quickly as possible, without retrieving dropped pegs. Before testing, a practice trial was conducted for each hand, followed by two consecutive test trials per hand, with the mean time recorded for analysis. The procedure was video‐recorded, and two independent analysts used a stopwatch to measure the time from first peg touch to last peg hitting the dish [23, 25]. If a participant paused after placing all pegs, retrieved fallen pegs, or deviated from instructions, the trial was discarded and repeated, with the reason documented. Any interruptions or errors, such as dropped pegs or talking, were noted. Additionally, if the time divergence between analysts exceeded 0.2431 s (based on the simple reaction time [22]), the trial was re‐reviewed until the discrepancy was ≤ 0.2431 s.

### 2.5 Randomization and Blinding

Randomization tables generated via a computer‐based algorithm assigned participants to two counterbalanced visits, each receiving a unique randomization ID that determined the sequence of active and control stimulation. These IDs and sequences were recorded in an Excel sheet and managed by the TPS operator, who administered TPS according to the predetermined order—though the operator was not blinded in this study.

Participants, lacking neuroscience or medical backgrounds, were blinded to the study hypothesis. They were informed that they would receive stimulation at two different targets during two visits, but they were not informed of the existence of a control condition (vertex stimulation). Before stimulation, both active and control targets were determined and marked during both visits to ensure the integrity of participants' blinding. To assess the effectiveness of the blinding, they were asked at the end of their second visit whether they expected a performance difference between visits.

Two blinded analysts independently processed behavioral data. They recorded NHPT completion times via video analysis, extracted SRTT reaction times from the computer program, and excluded outliers from the reaction time dataset.

### 2.6 Statistical Analysis

A linear mixed‐effects model was used to analyze the effects of time, stimulation condition, and their interaction, while accounting for potential confounding factors. Stimulation conditions (active vs. control), time points (T0–T5), and their interaction were set as fixed factors, with subject number as a random effect. The stimulation sequence (active‐control vs. control‐active) and baseline NHPT/SRTT performance were included as covariates. When significant main effects or interaction effects were found for NHPT or SRTT, post hoc tests were performed to examine between‐ and within‐group differences.

## 3 Results

### 3.1 Behavioral Outcomes

All 34 randomized participants completed the study, yielding analyzable behavioral data.

For left‐hand NHPT performance, the linear mixed‐effects model revealed a significant main effect of time (*F* = 10.872, *p* < 0.001), but no significant main effect of stimulation condition (active vs. control, *F* = 0.002, *p* = 0.967) or interaction effect (*F* = 0.244, *p* = 0.943). Post hoc paired *t*‐tests examined changes from baseline across post‐TPS time points for both active and control visits. During the active visit (rM1‐TPS), left‐hand NHPT performance significantly improved 10 to 40 min post‐TPS with medium to large effect sizes:

10 min: *t* = 3.29, *p*_corrected_ = 0.01, Cohen's *d* = 0.564.

20 min: *t* = 5.07, *p*_corrected_ < 0.001, Cohen's *d* = 0.869.

30 min: *t* = 4.71, *p*_corrected_ < 0.001, Cohen's *d* = 0.808.

40 min: *t* = 5.078, *p*_corrected_ < 0.001, Cohen's *d* = 0.871.

During the control visit (vertex‐TPS), similar significant improvements were observed between 10 and 40 min, with medium effect sizes:

10 min: *t* = 3.40, *p*_corrected_ = 0.01, Cohen's *d* = 0.582.

20 min: *t* = 3.37, *p*_corrected_ = 0.01, Cohen's *d* = 0.578.

30 min: *t* = 3.86, *p*_corrected_ < 0.001, Cohen's *d* = 0.662.

40 min: *t* = 3.54, *p*_corrected_ = 0.005, Cohen's *d* = 0.607.

**FIGURE 4:**
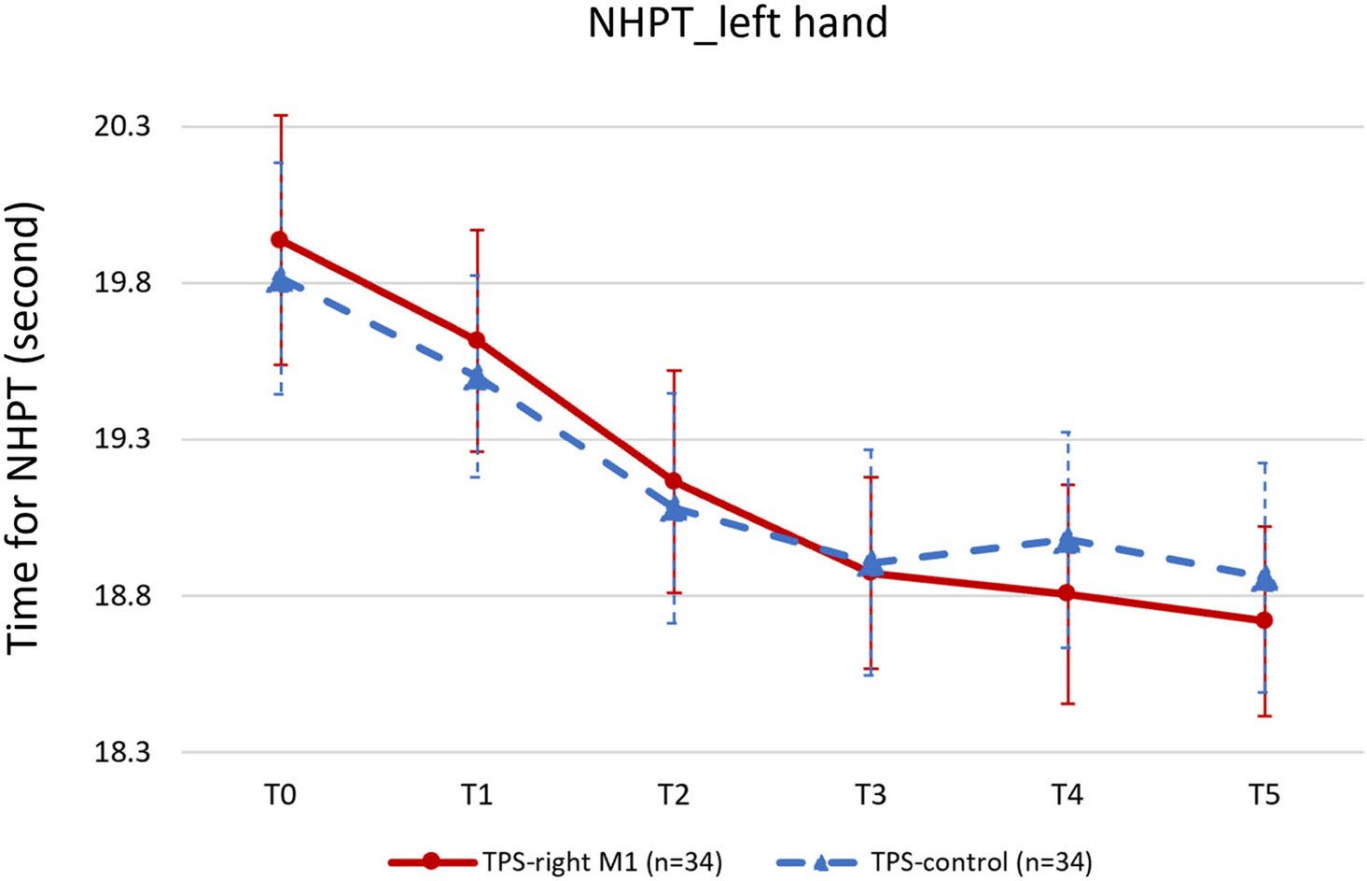
Changes in left‐hand Nine‐Hole Peg Test (NHPT) performance over time for active and control TPS. The dots and triangles indicate the mean time for NHPT, and the bars indicate the standard errors (SEs) for each session. T0: Baseline; T1: Immediately after TPS; T2: 10 min after TPS; T3: 20 min after TPS; T4: 30 min after TPS; T5: 40 min after TPS.

Therefore, rM1‐TPS did not significantly enhance NHPT performance compared to vertex‐TPS, although effect sizes were larger for rM1 stimulation between 20 and 40 min post‐TPS. Changes in left‐hand NHPT performance over time for both visits are shown in Figure 4 and Table 1.

**TABLE 1:** The left‐hand NHPT performance for both visits.

	Baseline	Immediately post‐TPS	10 min post‐TPS	20 min post‐TPS	30 min post‐TPS	40 min post‐TPS
rM1‐TPS visit	19.94 ± 0.40	19.61 ± 0.35 (*p*_corrected_ = 0.084)	19.16 ± 0.36* (*p*_corrected_ = 0.002, Cohen's *d* = 0.564)	18.87 ± 0.31* (*p*_corrected_ < 0.001, Cohen's *d* = 0.869)	18.80 ± 0.35* (*p*_corrected_ < 0.001, Cohen's *d* = 0.808)	18.72 ± 0.30* (*p*_corrected_ < 0.001, Cohen's *d* = 0.871)
Vertex‐TPS visit	19.81 ± 0.37	19.50 ± 0.32 (*p*_corrected_ = 0.144)	19.10 ± 0.37* (*p*_corrected_ = 0.002, Cohen's *d* = 0.582)	18.90 ± 0.36* (*p*_corrected_ = 0.002, Cohen's *d* = 0.578)	18.98 ± 0.35* (*p*_corrected_ < 0.001, Cohen's *d* = 0.662)	18.86 ± 0.37* (*p*_corrected_ = 0.001, Cohen's *d* = 0.607)

*Note:* Data is shown as mean ± SE. *p*‐values were calculated by using paired *t*‐tests to investigate the differences between the baseline.

* The significance level of corrected *p*‐values should be < 0.01 by using Bonferroni correction.

For SRTT performance, no significant main effects or interaction effects were observed during either visit. Changes in left‐hand SRTT performance over time for both visits are illustrated in the Supporting Information (Figure S1 and Table S1).

### 3.2 TPS Safety

Among the 34 participants, 25 (74%) in the rM1‐TPS visit and 25 (74%) in the vertex‐TPS visit reported side effects, with the most common ones being tingling and pressure sensation at the stimulation site. See details in the Table S2. All the symptoms resolved within 24 h without medication needed.

### 3.3 Integrity of the Blind

Participants were asked whether they thought they were expected to perform better during one of the two visits. Nine (27%) of the 34 participants believed they were expected to perform better during the rM1‐TPS visit, while 12 (35%) expected better performance during the vertex‐TPS visit; the remaining 13 participants (38%) reported uncertainty. A binary test confirmed that participants' expectations (rM1‐TPS visit vs. vertex‐TPS visit) were no better than chance (*p* = 0.664), demonstrating successful blinding of participants to the stimulation condition.

## 4 Discussion

This randomized, controlled, subject‐ and analyst‐blind crossover study assessed the feasibility of a single‐session TPS protocol and its modulatory effects on motor performance in healthy adults. The results confirm that delivering 1000 pulses of TPS to the right primary motor cortex is both feasible and safe, with no serious adverse events reported. No significant difference was observed between active and control stimulation, with left‐hand NHPT performance demonstrating larger effect sizes between 20 and 40 min post rM1‐TPS (Cohen's *d* = 0.808–0.871) than after vertex‐TPS (Cohen's *d* = 0.578–0.662). A post hoc power analysis indicated that a total sample size of 180 participants would be required to detect a significant interaction effect between stimulation condition and time, with an alpha of 0.05 and 80% power.

A recent study demonstrated immediate tremor amplitude reduction in PD patients following a single‐session TPS applied to the motor cortex contralateral to the most affected body side [15]. They found that active TPS significantly decreased the amplitude of resting tremors immediately after the stimulation, compared to the sham stimulation, with a large effect size (Cohen's *d* = 1.341). While promising, these results await replication. In any case, our study has several key differences from Manganotti et al.'s study [15] that may explain the differences in results. First, while Mangonotti et al. examined clinical populations and symptom changes, we investigated behavioral performance in healthy adults, making direct comparisons between these two studies difficult. Second, our protocol delivered 1000 pulses at the TMS‐defined hotspot, whereas Manganotti et al. administered 1500 pulses over the motor cortex without a detailed illustration of the targeting method or the size of the stimulation area. On the other hand, the potential dose‐dependent effect of TPS may partly explain their larger effect size. Third, methodological limitations in Manganotti et al.'s study may have influenced their results. Their non‐randomized trial treated 16 PD patients with active TPS first, followed by sham treatment for only nine patients, potentially introducing selection bias and blinding bias that could compromise reliability. Fourth, Manganotti et al. did not assess the time course of TPS's immediate effects on motor function. Our study is the first to investigate the short‐term behavioral effects of a single‐session TPS for up to 40 min post‐stimulation.

No significant improvements were detected in SSRT performance following either rM1‐TPS or vertex‐TPS. One possible explanation is that SSRT relies not only on sensory‐motor processing but also on attentional demand, which is closely linked to frontal cortex function [26]. Additionally, the ceiling effect observed in healthy participants may have limited detectable improvements.

Our study has several limitations. First, the washout period may have been insufficient. However, Manganotti et al. [15] found that the active TPS group demonstrated significantly greater improvement in tremor amplitude compared to the sham group immediately after TPS, rather than 24 h post‐stimulation. This suggests that a single session of TPS may modulate motor function more effectively than a placebo in the immediate period. In addition, several crossover trials on transcranial ultrasound stimulation also included a 24‐h washout period [27, 28]. Second, we used vertex stimulation as a control to maintain participant blinding, but this control stimulation may have induced a general stimulation effect, potentially introducing confounding factors into our results. Third, a single session of TPS may not be sufficient to demonstrate effectiveness on behavioral improvements. Most TPS studies have employed multiple sessions administered over 2–4 weeks [7, 9, 11, 13, 14]. Moreover, our unpublished data in MDD patients demonstrated greater clinical improvement with active TPS compared to sham treatment, particularly between weeks 2 and 4 of continued therapy.

Although no short‐term behavioral effects of M1‐TPS were observed when compared to vertex‐TPS, our findings illuminate potential directions for future TPS research. First, given the fact that a recent animal experiment has found real‐time neurovascular effects during TPS [29], future studies should investigate the online and short‐term effects of TPS using combined neurophysiological and neuroimaging assessments. Second, the inclusion of a sham control condition is essential to clarify the target‐specific modulatory effects of TPS. Third, the efficacy of multiple TPS sessions should be explored to determine if they induce stronger aftereffects.

## 5 Conclusion

A single session of rM1‐TPS does not significantly improve motor dexterity compared to vertex‐TPS. Both conditions improve performance from 10 to 40 min post‐stimulation. Future well‐powered studies that include a sham control condition and multiple stimulation sessions are needed to investigate the aftereffects of TPS using combined neurophysiological and neuroimaging approaches.

## Funding

This work was supported by the Mental Health Research Centre (Grant number: P0040786); the Strategic Hiring Scheme (Grant number: P0042417); and the Department of Rehabilitation Sciences (Grant number: P0043155 and P0055985) of the Hong Kong Polytechnic University.

## Ethics Statement

The study protocol was approved by the Human Subjects Ethics Sub‐committee of The Hong Kong Polytechnic University (Reference No: HSEARS20240319001‐03).

## Consent

All the included participants were fully informed of the experimental procedures and signed the written informed consent before the experiment. The study was conducted in accordance with the Declaration of Helsinki.

## Conflicts of Interest

The authors declare no conflicts of interest.

## Supporting information


**Figure S1:** Changes in left‐hand Simple Reaction Time Task (SRTT) performance over time for active and control TPS. The dots and triangles indicate the mean reaction time for SRTT, and the bars indicate the standard errors (SEs) for each session. T0: baseline; T1: immediately after TPS; T2: 10 min after TPS; T3: 20 min after TPS; T4: 30 min after TPS; T5: 40 min after TPS.
**Table S1:** The left‐hand Simple Reaction Time Task (SRTT) performance for both visits. Data is shown as mean ± SE. *p*‐values were calculated by using the Wilcoxon test to investigate the differences between the baseline. *The significance level of corrected *p*‐values should be < 0.01 by using Bonferroni correction.
**Table S2:** Adverse events occurred during the experimental period. rM1‐TPS: transcranial pulse stimulation over the right primary motor cortex; Vertex‐TPS: transcranial pulse stimulation over the vertex.

## Data Availability

The data is available from the corresponding author upon reasonable request.
