# Supplementary material for: Effects of Transcranial Pulse Stimulation of the Primary Motor Cortex on Motor Performance in Healthy Adults: A Randomized Crossover Pilot Study
Source: CNS Neurosci Ther. 2025 Dec 20;31(12):e70711. doi: 10.1002/cns.70711 (PMC12717462; doi:10.1002/cns.70711)
Supplement: Supplementary file 1 — Figure S1: Changes in left‐hand Simple Reaction Time Task (SRTT) performance over time for active and control TPS. The dots and triangles indicate the mean reaction time for SRTT, and the bars indicate the standard errors (SEs) for each session. T0: baseline; T1: immediately after TPS; T2: 10 min after TPS; T3: 20 min after TPS; T4: 30 min after TPS; T5: 40 min after TPS. Table S1: The left‐hand Simple Reaction Time Task (SRTT) performance for both visits. Data is shown as mean ± SE. p‐values were calculated by using the Wilcoxon test to investigate the differences between the baseline. *The significance level of corrected p‐values should be < 0.01 by using Bonferroni correction. Table S2: Adverse events occurred during the experimental period. rM1‐TPS: transcranial pulse stimulation over the right primary motor cortex; Vertex‐TPS: transcranial pulse stimulation over the vertex. [file CNS-31-e70711-s001.docx]

**Supplementary Figure 1.** Changes in left-hand Simple Reaction Time Task (SRTT) performance over time for active and control TPS. The dots and triangles indicate the mean reaction time for SRTT, and the bars indicate the standard errors (SEs) for each session. T0: baseline; T1: immediately after TPS; T2: 10 minutes after TPS; T3: 20 minutes after TPS; T4: 30 minutes after TPS; T5: 40 minutes after TPS.


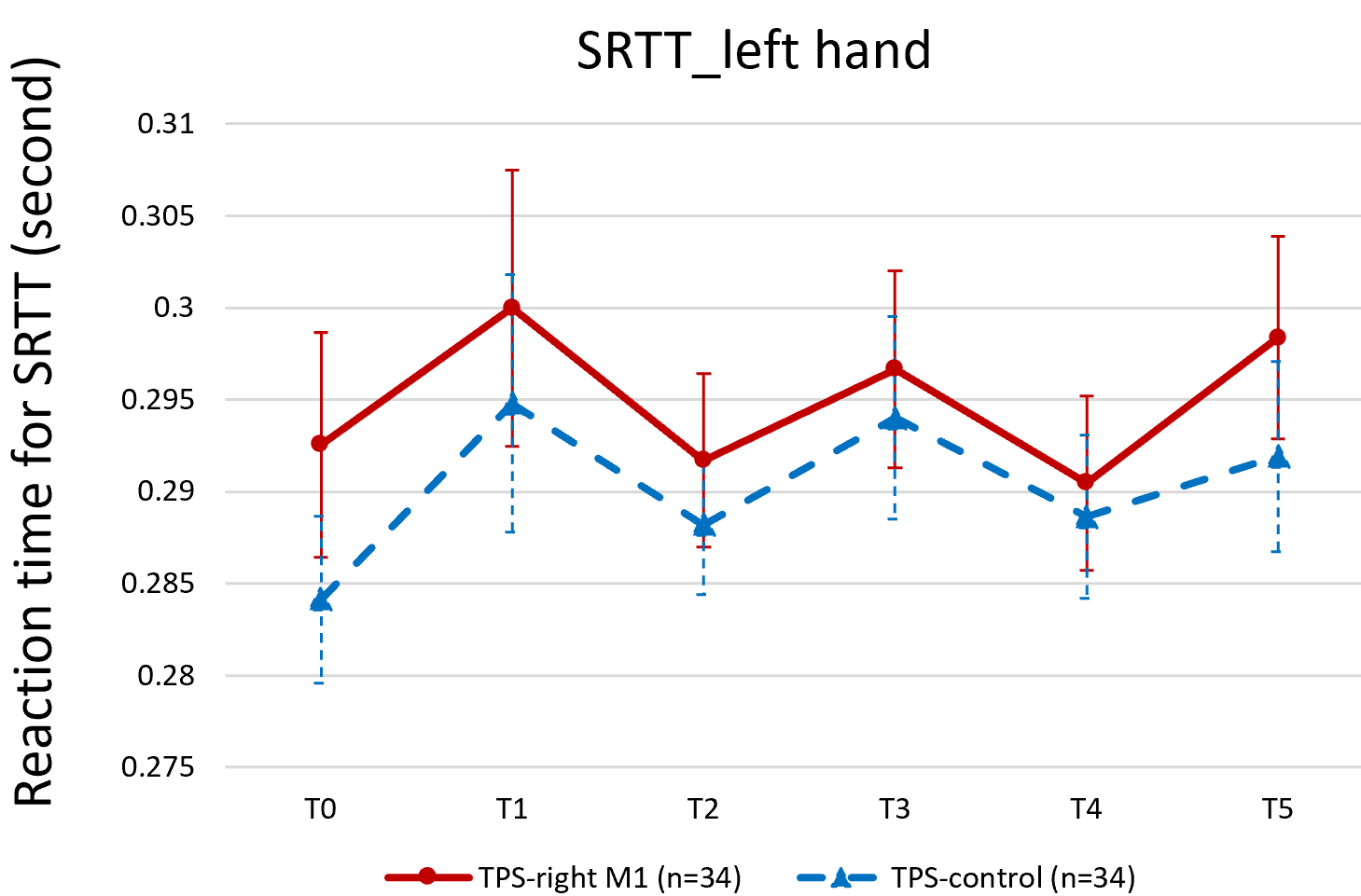


**Supplementary Table 1.** The left-hand Simple Reaction Time Task (SRTT) performance for both visits. Data is shown as mean ± SE. p-values were calculated by using the Wilcoxon test to investigate the differences between the baseline. *: The significance level of corrected p-values should be < 0.01 by using Bonferroni correction.

|  | Baseline | Immediately post TPS | 10min post TPS | 20min post TPS | 30min post TPS | 40min post TPS |
| --- | --- | --- | --- | --- | --- | --- |
| rM1-TPS visit | 0.292543±  0.006101 | 0.299988±  0.007517 (p_corrected_=0.447) | 0.291702±  0.004717 (p_corrected_=0.544) | 0.29666±  0.00536 (p_corrected_=0.197) | 0.290466±  0.004726 (p_corrected_=0.993) | 0.298368±  0.005513 (p_corrected_=0.169) |
| Vertex-TPS visit | 0.284121±  0.004525 | 0.294794±  0.007007 (p_corrected_=0.071) | 0.288196±  0.003787 (p_corrected_=0.135) | 0.294014±  0.005501 (p_corrected_=0.077) | 0.288632±  0.004436 (p_corrected_=0.263) | 0.291919±  0.00518 (p_corrected_=0.103) |

**Supplementary Table 2.** Adverse events occurred during the experimental period. rM1-TPS: transcranial pulse stimulation over the right primary motor cortex; Vertex-TPS: transcranial pulse stimulation over the vertex.

|  | Number of participants reporting each adverse event during stimulation (%) | | |
| --- | --- | --- | --- |
|  | rM1-TPS | Vertex-TPS | |
| Tingling | 8 (24%) | 8 (24%) | |
| Pressure sensation | 3 (9%) | 5 (15%) | |
| pain | 3 (9%) | 5 (15%) | |
| numbness | 2 (6%) | 3 (9%) | |
| Striking sensation | 2 (6%) | 1 (3%) | |
| Vibration in the ears | 2 (6%) | 2 (6%) | |
| Unpleasant noise | 1 (3%) | 1 (3%) | |
| Dizziness | 1 (3%) | 1 (3%) | |
| Decreased hearing | 1 (3%) | NA | |
| Headache | 1 (3%) | NA | |
| Pain around the left ear | NA | 2 (6%) | |
| Irritation | NA | 1 (3%) | |
|  | Number of participants reporting each adverse event after stimulation (%) | | |
| Fatigue | 1 (3%) | | NA |
| Decreased hearing | 1 (3%) | | NA |
| Dizziness | 1 (3%) | | NA |
| Headache | 1 (3%) | | NA |
| Sourness in the arm and hand | 1 (3%) | | NA |
| Pain around the left ear | NA | | 1 (3%) |
| Muscle tightness in the right face | NA | | 1 (3%) |
